# Eight Common Genetic Variants Associated with Serum DHEAS Levels Suggest a Key Role in Ageing Mechanisms

**DOI:** 10.1371/journal.pgen.1002025

**Published:** 2011-04-14

**Authors:** Guangju Zhai, Alexander Teumer, Lisette Stolk, John R. B. Perry, Liesbeth Vandenput, Andrea D. Coviello, Annemarie Koster, Jordana T. Bell, Shalender Bhasin, Joel Eriksson, Anna Eriksson, Florian Ernst, Luigi Ferrucci, Timothy M. Frayling, Daniel Glass, Elin Grundberg, Robin Haring, Åsa K. Hedman, Albert Hofman, Douglas P. Kiel, Heyo K. Kroemer, Yongmei Liu, Kathryn L. Lunetta, Marcello Maggio, Mattias Lorentzon, Massimo Mangino, David Melzer, Iva Miljkovic, MuTHER Consortium, Alexandra Nica, Brenda W. J. H. Penninx, Ramachandran S. Vasan, Fernando Rivadeneira, Kerrin S. Small, Nicole Soranzo, André G. Uitterlinden, Henry Völzke, Scott G. Wilson, Li Xi, Wei Vivian Zhuang, Tamara B. Harris, Joanne M. Murabito, Claes Ohlsson, Anna Murray, Frank H. de Jong, Tim D. Spector, Henri Wallaschofski

**Affiliations:** 1 Department of Twin Research and Genetic Epidemiology, King's College London, London, United Kingdom; 2 Interfaculty Institute for Genetics and Functional Genomics, University of Greifswald, Greifswald, Germany; 3 Department of Internal Medicine, Erasmus MC Rotterdam, Rotterdam, The Netherlands; 4 Netherlands Consortium of Healthy Ageing, Rotterdam, The Netherlands; 5 Genetics of Complex Traits, Peninsula Medical School, University of Exeter, Exeter, United Kingdom; 6 Wellcome Trust Centre for Human Genetics, University of Oxford, Oxford, United Kingdom; 7 Department of Internal Medicine, Institute of Medicine, Sahlgrenska Academy, University of Gothenburg, Gothenburg, Sweden; 8 Sections of General Internal Medicine, Preventive Medicine, Cardiology and Endocrinology, Diabetes and Nutrition, Department of Medicine, Boston University School of Medicine, Boston, Massachusetts, United States of America; 9 Laboratory for Epidemiology, Demography, and Biometry, National Institute on Aging, Bethesda, Maryland, United States of America; 10 Section of Endocrinology, Diabetes, and Nutrition, Claude D. Pepper Older Americans Independence Center, Boston University School of Medicine, Boston, Massachusetts, United States of America; 11 Clinical Research Branch, National Institute on Aging, Baltimore, Maryland, United States of America; 12 Wellcome Trust Sanger Institute, Hixton, United Kingdom; 13 Institute for Clinical Chemistry and Laboratory Medicine, University of Greifswald, Greifswald, Germany; 14 Department of Epidemiology, Erasmus MC, Rotterdam, The Netherlands; 15 Hebrew Senior Life Institute for Aging Research and Harvard Medical School, Boston, Massachusetts, United States of America; 16 Center of Pharmacology and Experimental Therapeutics, Department of Pharmacology, University of Greifswald, Greifswald, Germany; 17 Department of Epidemiology and Prevention, Wake Forest University Health Sciences, Winston-Salem, North Carolina, United States of America; 18 Department of Biostatistics, Boston University School of Public Health, Boston, Massachusetts, United States of America; 19 Department of Internal Medicine and Biomedical Sciences, Section of Geriatrics, University of Parma, Parma, Italy; 20 Department of Epidemiology, University of Pittsburgh, Pittsburg, Pennsylvania, United States of America; 21 Department of Genetic Medicine and Development, University of Geneva Medical School, Geneva, Switzerland; 22 Department of Psychiatry, VU University Medical Center, Amsterdam, The Netherlands; 23 The National Heart Lung and Blood Institute's Framingham Heart Study, Framingham, Massachusetts, United States of America; 24 Institute for Community Medicine, University of Greifswald, Greifswald, Germany; 25 Department of Endocrinology and Diabetes, Sir Charles Gairdner Hospital, Nedlands, Australia; 26 School of Medicine and Pharmacology, University of Western Australia, Nedlands, Australia; 27 Molecular Medicine – Computational Biology, Pfizer Worldwide R&D, Groton, Connecticut, United States of America; Georgia Institute of Technology, United States of America

## Abstract

Dehydroepiandrosterone sulphate (DHEAS) is the most abundant circulating steroid secreted by adrenal glands—yet its function is unknown. Its serum concentration declines significantly with increasing age, which has led to speculation that a relative DHEAS deficiency may contribute to the development of common age-related diseases or diminished longevity. We conducted a meta-analysis of genome-wide association data with 14,846 individuals and identified eight independent common SNPs associated with serum DHEAS concentrations. Genes at or near the identified loci include *ZKSCAN5* (rs11761528; p = 3.15×10^−36^), *SULT2A1* (rs2637125; p = 2.61×10^−19^), *ARPC1A* (rs740160; p = 1.56×10^−16^), *TRIM4* (rs17277546; p = 4.50×10^−11^), *BMF* (rs7181230; p = 5.44×10^−11^), *HHEX* (rs2497306; p = 4.64×10^−9^), *BCL2L11* (rs6738028; p = 1.72×10^−8^), and *CYP2C9* (rs2185570; p = 2.29×10^−8^). These genes are associated with type 2 diabetes, lymphoma, actin filament assembly, drug and xenobiotic metabolism, and zinc finger proteins. Several SNPs were associated with changes in gene expression levels, and the related genes are connected to biological pathways linking DHEAS with ageing. This study provides much needed insight into the function of DHEAS.

## Author Summary

Dehydroepiandrosterone sulphate (DHEAS), mainly secreted by the adrenal gland, is the most abundant circulating steroid in humans. It shows a significant physiological decline after the age of 25 and diminishes about 95% by the age of 85 years, which has led to speculation that a relative DHEAS deficiency may contribute to the development of common age-related diseases or diminished longevity. Twin- and family-based studies have shown that there is a substantial genetic effect with heritability estimate of 60%, but no specific genes regulating serum DHEAS concentration have been identified to date. Here we take advantage of recent technical and methodological advances to examine the effects of common genetic variants on serum DHEAS concentrations. By examining 14,846 Caucasian individuals, we show that eight common genetic variants are associated with serum DHEAS concentrations. Genes at or near these genetic variants include *BCL2L11*, *ARPC1A*, *ZKSCAN5*, *TRIM4*, *HHEX*, *CYP2C9*, *BMF*, and *SULT2A1*. These genes have various associations with steroid hormone metabolism—co-morbidities of ageing including type 2 diabetes, lymphoma, actin filament assembly, drug and xenobiotic metabolism, and zinc finger proteins—suggesting a wider functional role for DHEAS than previously thought.

## Introduction

Dehydroepiandrosterone sulphate (DHEAS), mainly secreted by the adrenal gland, is the most abundant circulating steroid in humans. It acts as an inactive precursor which is converted initially into DHEA and thereafter into active androgens and estrogens in peripheral target tissues [1]. In humans the serum concentration of circulating DHEAS is 100- to 500-fold or 1000 to 10,000 higher than that of testosterone and estradiol respectively. Unlike DHEA, which is swiftly cleared from the circulation and shows diurnal variation, serum DHEAS concentrations are stable and facilitate accurate measurement and diagnosis of pathology [2].

DHEAS is distinct from the other major adrenal steroids (cortisol and aldosterone) in showing a significant physiological decline after the age of 25 and diminishes about 95% by the age of 85 years [3]. This age-related decline has led to speculation that a relative DHEAS deficiency may contribute to the development of common age-related diseases or diminished longevity [4], [5]. Low DHEAS concentrations are possibly associated with increased insulin resistance [6], [7] and hypertension [8], but not with incident metabolic syndrome [9]. It is strongly associated with osteoporosis in women [10], [11] but not in men [12]. Concurrent change in DHEAS tracks with declines in gait speed, modified mini-mental state examination score (3MSE), and digit symbol substitution test (DSST) in very old women but not in men [13]. Low circulating DHEAS is also strongly associated with cardiovascular disease and mortality in men [14]–[18] but not in women [19]. A recent 15-year follow-up study showed that DHEAS was negatively related to all-cause, all cancers, and other medical mortality, whereas high DHEAS concentrations were protective [20]. This has led to its widespread and uncontrolled use as a controversial anti-ageing and sexual performance supplement in the USA and other western countries without any clear data about efficacy, potential risks or benefits [21].

Despite these observations, the physiological function of DHEAS and its importance in maintaining health are poorly understood. Although previous twin [22], [23] and family-based studies [24], [25] have shown that there is a substantial genetic effect with a heritability estimate of 60% [22], no specific genes regulating serum DHEAS concentration in healthy individuals have been identified to date. Therefore, the current study meta-analyzed the results of genome-wide association studies (GWAS) performed in a total of 14,846 individuals from seven cohorts to identify common genetic variants associated with serum DHEAS concentrations. The findings not only advance understanding of how serum DHEAS concentration is regulated by genes but also provide clues as to its mechanism of action as well as Mendelian randomisation principles [26].

## Results

We carried out a meta-analysis of 8,565 women and 6,281 men of European origin from collaborating studies: TwinsUK (n = 4,906), Framingham Heart Study (FHS) (n = 3,183), SHIP (n = 1,832), Rotterdam Study (RS1) (n = 1,597), InCHIANTI (n = 1,182), Health ABC (n = 1,222), and GOOD (n = 924). Serum samples were collected either after overnight fasting or non-fasting in each cohort and DHEAS was measured by either immunoassay or liquid chromatography tandem mass spectrometry (LC-MS/MS) methods (Table 1). Mean age differed across the cohorts from 19 to 74 years in men and 50 to 74 years in women and corresponding mean DHEAS concentrations varied from 1.20 to 7.05 µmol/L (Table 1).

**Table 1:** Descriptive statistics of serum levels of DHEAS (µmol/L) for each cohort.

Males									
Cohort	Assay	Mean Age (Range)	Mean	SD	Median	Min	Max	Range	n
***RS1***	Immunoassay	69 (55–98)	4.34	2.88	3.70	0.10	23.08	22.98	*740*
***SHIP***	Immunoassay	51 (20–79)	1.90	1.21	1.64	0.31	8.90	8.59	1832
***FHS***	Immunoassay	51 (25–80)	7.05	5.07	5.35	0.27	29.86	29.59	*1571*
***GOOD***	MassSpec	19 (18–20)	6.31	2.33	6.04	1.27	15.10	13.83	924
***InCHIANTI***	Immunoassay	67 (23–94)	3.16	2.98	2.25	0.02	33.06	33.04	*518*
***HABC***	Immunoassay	74 (69–80)	1.58	1.12	1.40	0.00	9.93	9.93	696
								*n Total*	*6281*

Females									
Cohort	Assay	Mean Age (Range)	Mean	SD	Median	Min	Max	Range	n
***TwinsUK I***	Immunoassay	50 (17–82)	3.95	2.47	3.40	0.20	19.30	19.10	2541
***TwinsUK II***	Immunoassay	50 (16–82)	4.21	2.79	3.60	0.10	22.30	22.20	*2365*
***RS1***	Immunoassay	69 (55–98)	2.65	2.03	2.11	0.01	13.61	13.60	*857*
***FHS***	Immunoassay	51 (22–77)	3.84	2.96	3.02	0.30	21.01	20.71	1612
***InCHIANTI***	Immunoassay	68 (21–95)	2.69	2.35	1.96	0.04	15.29	15.25	*664*
***HABC***	Immunoassay	74 (69–80)	1.20	0.88	0.97	0.00	5.59	5.59	526
								*n Total*	*8565*

Each cohort performed GWA tests for log transformed DHEAS on ∼2.5 million imputed single nucleotide polymorphisms (SNPs) in men and women separately with adjustment for age, and additionally for age and sex for those cohorts who had data in both men and women. Then Z-scores from each cohort were pooled for the meta-analysis at each SNP.

In all our individual GWAS, λ_GC_, which is defined as the median χ^2^ (1 degree of freedom) association statistic across SNPs divided by its theoretical median under the null distribution [27], ranged from 0.984 to 1.023, indicating that there was no population stratification or it was very minor. Further, we corrected for population stratification by applying the genomic control method [27]; the λ_GC_ in the meta-analysis is 1.017. In addition, the effect direction was consistent across all the cohorts and there is no between-study heterogeneity as indicated by I^2^ ranging between 0 and 0.12 (Table 2).

**Table 2:** SNPs associated with serum DHEAS concentrations: genome-wide results of meta-analysis of men and women combined.

SNP	Chr	Position in base pair	Freq	Effect Allele	Beta (SE)*	P value	I^2^ index¶	Effect direction in each study	Gene	Distance to the gene
**Discovery meta-analysis**										
rs11761528	7	98956737	0.08	T	−0.16 (0.01)	3.15×10^−36^	0.12	−−−−−−−−	*ZKSCAN5*	intron
rs2637125	19	53093705	0.15	A	−0.09(0.01)	2.61×10^−19^	0.00	−−−−−−−−	*SULT2A1*	12 kb
rs7181230	15	38148033	0.33	G	0.05(0.01)	5.44×10^−11^	0.00	++++++++	*BMF*	23 kb
rs2497306	10	94475191	0.49	C	−0.04(0.01)	4.64×10^−9^	0.00	−−−−−−−−	*HHEX*	25 kb
rs2185570	10	96741260	0.13	C	−0.06(0.01)	2.29×10^−8^	0.00	−−−−−−−−	*CYP2C9*	−2 kb
**Conditional analysis**										
rs740160§	7	98795816	0.05	T	0.15 (0.02)	1.56×10^−16^	0.02	++++++++	*ARPC1A*	intron
rs17277546§	7	99327507	0.05	A	−0.11 (0.02)	4.50×10^−11^	0.00	−−−−−−−−	*TRIM4;CYP3A43*	3′UTR
rs6738028§	2	111665798	0.40	G	−0.04 (0.01)	1.72×10^−8^	0.00	−−−−−−−−	*BCL2L11*	−62 kb

*Beta was expressed as natural log changes in serum DHEAS concentration in µmol/L per copy of the risk allele.

**¶:** index for between-study heterogeneity: 0.25 – low, 0.50 – moderate and 0.75 – high heterogeneity.

**§:** pre-conditional p values were 0.612, 1.90×10^−26^, and 1.94×10^−7^ for rs740160, rs17277546, and rs6738028, respectively.

**Figure 1:**
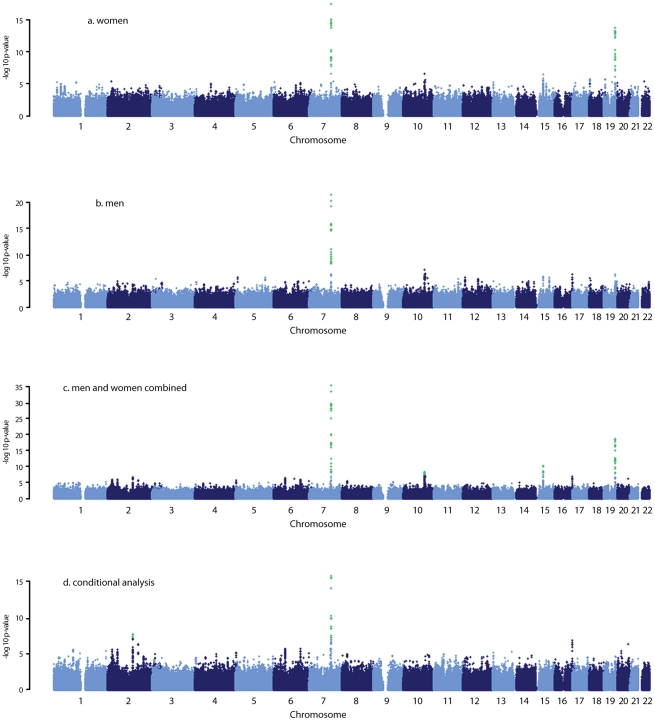
Manhattan plots for the genome-wide meta-analysis results. Green dots indicate the SNPs with p<5×10^−8^.

We found 44 SNPs were associated with serum DHEAS concentrations in men at conventional genome-wide significance (p<5×10^−8^), which are all located on chromosome 7q22.1 (Figure 1B; Table S1). All these SNPs except for three were significant in women (Figure 1A; Table S1). In addition, 19 SNPs located on chromosome 19q13.3 were found in women to be associated with serum DHEAS concentrations with p<5×10^−8^. In the sex-combined meta-analysis, the significance became stronger for all these SNPs (Figure 1C; Table S1). Further, we found 8 SNPs located on chromosome 10q23.33 which represents two regions more than 2 MB apart, 12 SNPs on chromosome 15q15.1, and in addition, 4 SNPs on chromosome 19q13.3 were associated with serum DHEAS concentrations with p<5×10^−8^. Together we found a total of 87 SNPs associated with serum DHEAS concentrations with p<5×10^−8^, representing five chromosomal regions of less than 1 Mb each (Table S1).

The most significantly associated SNPs in each of these five regions are presented in Table 2. The minor allele of rs11761528 (p = 3.15×10^−36^) on chromosome 7q22.1, rs2637125 (p = 2.61×10^−19^) on chromosome 19q13.3, and rs2497306 (p = 4.6×10^−9^) and rs2185570 (p = 2.29×10^−8^) on chromosome 10q22.33 (more than 2 Mb apart), were negatively associated with DHEAS concentrations. In comparison, the minor allele of rs7181230 (p = 5.44×10^−11^) on chromosome 15q15.1 was positively associated with serum DHEAS concentrations. Based on the HapMap3 release2 CEU data, the significant 87 SNPs from within the five regions have low pair-wise r^2^, indicating potentially multiple independent signals. To verify this, we performed a conditional meta-analysis with adjustment for the five most significant SNPs plus age and sex in each cohort.

After this adjustment, all other SNPs on chromosome 10, 15, and 19 became non-significant (Figure 1D). However, on chromosome 7, we found two independent signals; one defined by rs11761528 and a second located 370 kb upstream in the 3′ UTR of the *TRIM4* and *CYP3A43* genes (rs17277546, p = 4.50×10^−11^). Furthermore, we identified two additional significant loci associated with DHEAS, one on chromosome 2q13 (rs6738028, p = 1.72×10^−8^), and another on chromosome 7 within the *ARPC1A* gene (rs740160 located 161 kb downstream of rs11761528, p = 1.56×10^−16^) (Table 2; Figure 1D). In total, we found eight independent SNPs associated with serum DHEAS concentrations at conventional genome-wide significant level (p<5×10^−8^) (Table 2). The effect was consistently in the same direction across all cohorts (Table 2). No heterogeneity among cohorts was observed (Table 2). These SNPs together explained ∼4% of the total and ∼7% of genetic variance of serum DHEAS concentrations (based on TwinsUK data). To further look at whether the magnitude of these genetic association varies with age, we carried out an interaction analysis between age and each of these 8 SNPs on serum DHEAS concentrations by including an interaction term of age×SNP in the linear regression model in each cohort and then meta-analyzed the results. We found that there was no significant interaction between age and each of these SNPs (all p values≥0.05).

**Figure 2:**
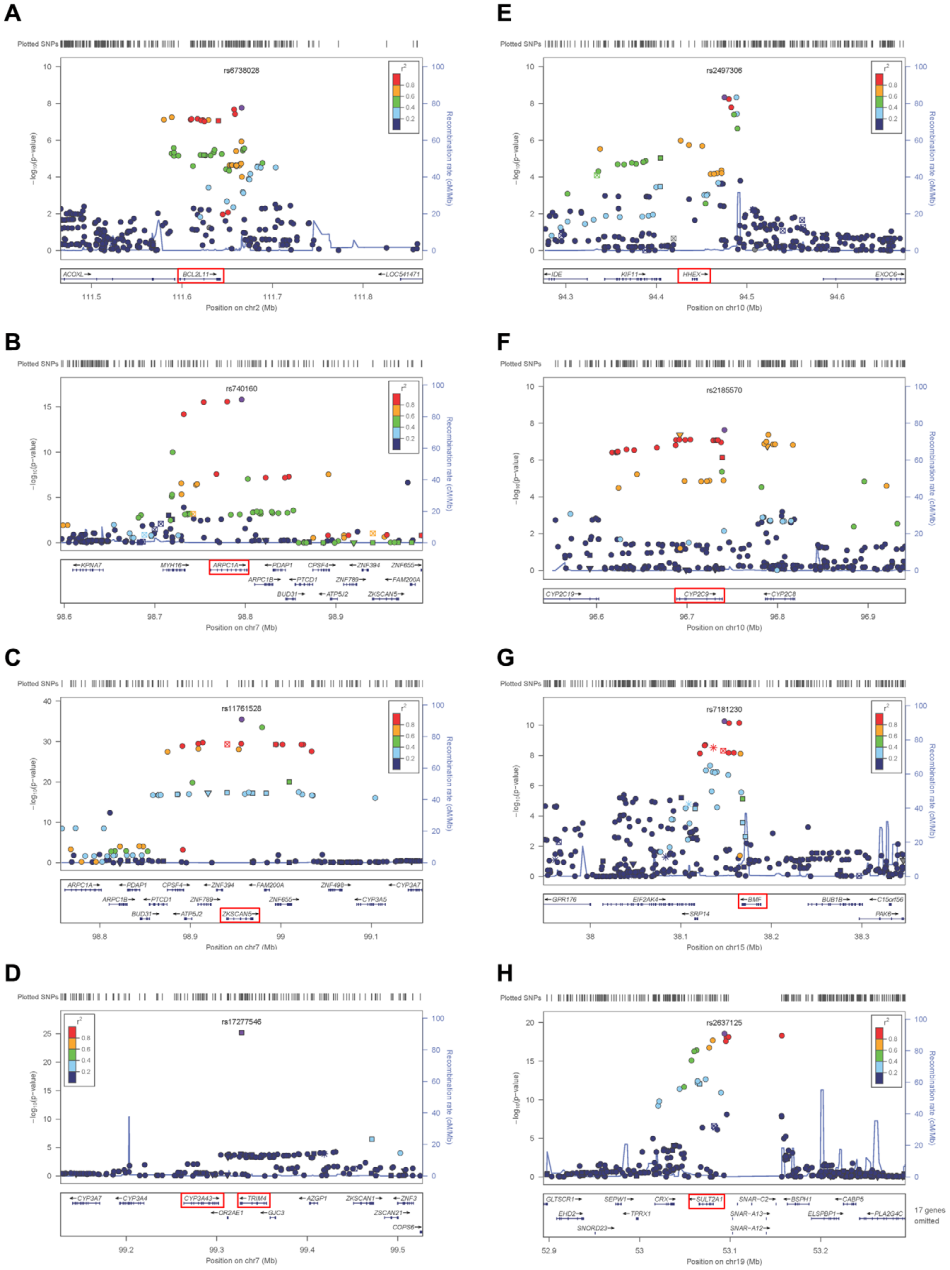
Regional linkage disequilibrium plots. For rs6738028 (A), rs740160 (B), rs11761528 (C), rs17277546 (D), rs2497306 (E), rs2185570 (F), rs7181230 (G), and rs2637125 (H). Note: p values from the conditional analysis were used for (a) and (b), both of them became genome-wide significant in the conditional analysis. Annotation key: ▴ - framestop or splice; ▾ - NonSynonymous; ▪ - Synonymous or UTR; •- nothing; *- TFBScons; 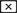 -MCS44 Placental.

The genes at, or near the identified SNPs, include *BCL2L11* on chromosome 2, *ZKSCAN5*, *ARPC1A*, *TRIM4* and *CYP3A43* on chromosome 7, *HHEX* and *CYP2C9* on chromosome 10, *BMF* on chromosome 15, and *SULT2A1* on chromosome 19 (Figure 2). To explore the potentially functional impacts and likely genetic mechanisms, we used two resources: Genome-wide expression data from the Multiple Tissue Human Expression Resource (MuTHER) [28] (http://www.muther.ac.uk/) based on ∼777 unselected UK twins sampled for skin, adipose tissue, and lymphoblastoid cell lines (LCLs) (more details in Text S1); and published gene expression data in human liver [29]. We found that 3 DHEAS-associated SNPs were clearly associated with the related gene expression levels in at least one tissue after accounting for multiple testing (Table 3). These specific transcript associations provide further evidence for the likely functional gene at each locus.

**Table 3:** Association between DHEAS-associated SNPs and related gene expression levels in different human tissues.

Gene	Chr	SNP (effect allele)	Position	LCL* (n = 777)		Adipose tissue* (n = 776)		Skin tissue* (n = 667)		Liver tissue¶ (n = 427)
				Beta (SE)	P value	Beta (SE)	P value	Beta (SE)	P value	P value
*BCL2L11*	2	rs6738028 (G)	111665798	0.07 (0.02)	0.0003	0.02 (0.005)	0.001	−0.00004 (0.005)	0.99	Not available
*TRIM4*	7	rs17277546 (A)	99327507	0.15 (0.04)	0.0001	0.13(0.04)	0.002	0.10(0.04)	0.01	Not available
*SULT2A1*	19	rs2637125 (A)/rs2547231**	53093705	0.0006 (0.007)	0.93	−0.009(0.007)	0.19	0.02(0.007)	0.01	2.16×10^−11^

*from MuTHER consortium and beta (SE) were from linear regression modelling; LCL – lymphoblastoid cell lines.

**¶:** from reference 27 and effect size was not reported.

**p value in liver expression is for rs2547231, data is not available for rs2637125, but two SNPs are in strong LD (r^2^ = 0.658).

Further, we carried out gene ontology and pathway analyses using a gene set enrichment analysis (GSEA) approach in MAGENTA [30] which consists of four main steps: First, DNA variants, e.g. SNP, are mapped onto genes. Second, each gene is assigned a gene association score that is a function of its regional SNP association p-values. Third, confounding effects on gene association scores are identified and corrected for, without requiring genotype data. Fourth, a GSEA-like statistical test is applied to predefined biologically relevant gene sets to determine whether any of the gene sets are enriched for highly ranked gene association scores compared to randomly sampled gene sets of identical size from the genome. More details of these four steps are described in the method section. In this analysis, we identified three pathways which passed our significance threshold (false discovery rate (FDR)<0.05); xenobiotic metabolism with FDR = 0.001 (pathway database: KEGG and Ingenuity), retinoid X receptor (RXR) function with FDR = 0.003 (pathway database: Ingenuity), and linoleic acid metabolism with FDR = 0.02 (pathway database: KEGG) (Figure S1). The top significant genes with p<5.0×10^−8^ include *CYP3A4*, *CYP3A43*, *CYP3A5*, and *CYP3A7* on chromosome 7, and *CYP2C8* and *CYP2C9* on chromosome 10 for all three pathways, and *SULT2A1* for RXR pathway. The best index SNPs are rs17277546 for *CYP3A4* and *CYP3A43*, rs4646450 for *CYP3A5* and *CYP3A7*, rs2185570 for *CYP2C9*, rs11572169 for *CYP2C8*, and rs2637125 for *SULT2A1*. The full list of the genes in each of the three pathways and the best index SNPs for each gene are listed in Table S2. Three SNPs – rs17277546, rs2185570, and rs2637125 are the DHEAS-associated SNPs found in our meta-analysis. Both rs4646450 and rs11572169 were associated with DHEAS with p values of 8.8×10^−17^ and 4.8×10^−8^, respectively, but become non-significant in the conditional meta-analysis because rs4646450 is in linkage disequilibrium (LD, r^2^ = 0.429) with rs11761528 which is the most significant DHEAS-associated SNP while rs11572169 is in high LD (r^2^ = 0.778) with rs2185570. Intriguingly, two pathways - xenobiotic metabolism and linoleic acid metabolism, have been linked to ageing in model organisms [31]–[36].

## Discussion

This is the first meta-analysis of GWA studies on serum DHEAS in 14,846 Caucasian subjects. We found 8 common SNPs that implicate nearby genes that are independently associated with serum DHEAS concentrations and provide clues to its role in ageing.

Among the genes identified, *SULT2A1*, a specialized sulpho-transferase which converts DHEA to DHEAS in the adrenal cortex, is an obvious candidate gene [3]. *SULT2A1* has a broad substrate specificity, which includes conversion of pregnenolone, 17α-hydroxypregnenolone, and DHEA to their respective sulphated products [37]. Once sulphated by *SULT2A1*, pregnenolone and 17α-hydroxypregnenolone are no longer available as substrates for *HSD3B2*. Therefore, *SULT2A1* sulphation of pregnenolone and 17α-hydroxypregnenolone removes these substrates from the mineralocorticoid and glucocorticoid biosynthetic pathways. This suggests that high levels of *SULT2A1* would ensure the formation of DHEAS [3].

Variation in *SULT2A1* expression has previously been associated with variation of DHEAS concentration [38]. The *SULT2A1* gene is predominantly expressed in the adrenal cortex and to a lesser extent in the liver. We found that rs2547231 (p = 1.76×10^−17^), located 12 kb downstream of *SULT2A1*, was strongly associated with expression levels of *SULT2A1* in human liver tissues. Although this SNP is not the most strongly associated with serum DHEAS, it is itself in strong LD with the most significant SNP rs2637125 (r^2^ = 0.658). However, we did not find a significant association with *SULT2A1* expression levels in LCL, skin, and adipose tissues, suggesting a tissue specific effect. The *SULT2B1b* is also reported to play a role in sulphation of DHEA, but in comparison, the strongest signal from that genomic region was rs10417472 with a p = 0.06. In contrast, enzymatic removal of the sulphate group from DHEAS to form DHEA is performed by steroid sulphatase gene (*STS*), but that gene is on the X chromosome and so was not assessed in this meta-analysis.

*CYP2C9* is an important cytochrome P450 enzyme, accounts for approximately 17–20% of the total P450 content in human liver, and catalyzes many reactions involved in drug metabolism as well as synthesis of cholesterol, steroids and other lipids [39]. We found that rs2185570 located in the *CYP2C9* gene region is associated with serum DHEAS concentrations. This SNP is in strong LD with rs4086116 and rs4917639 (r^2^ = 0.67 for both) which were found to be associated with acenocoumarol [40] and warfarin maintenance dosage [41] respectively in recent GWAS.

Two other cytochrome P450 enzymes – *CYP11A1* and *CYP17A1*, are two important enzymes which are required in the synthesis of DHEAS in the adrenal gland [3], however, the strongest signals in the genomic region were rs2930306 with p = 0.29 for *CYP11A1* and rs4919686 with p = 0.04 for *CYP17A1*.

The decline in serum DHEAS concentrations with increasing age has been proposed as a putative biomarker of ageing [21]. We found that two putative ageing genes – *BCL2L11* and *BMF* [42] are associated with serum DHEAS concentrations. Both of them encode proteins which belong to the *BCL2* family and act as anti- or pro-apoptotic regulators that are involved in a wide variety of cellular activities. *BCL2L11* has been implicated in chronic lymphocytic leukaemia (rs17483466, *P* = 2.36×10^−10^) [43] and follicular lymphoma (rs3789068, P for trend = 0.0004) [44]. The DHEAS-associated SNP rs6738028 is not however one of the same SNPs associated with lymphocytic leukaemia and follicular lymphoma nor is it in LD with them. Nevertheless, rs6738028 is strongly associated with *BCL2L11* gene expression levels in both LCL and adipose tissues, suggesting its putative regulatory role. There is relatively little data on the human *BMF* gene or the protein product, but *Bmf* ^−/−^ mice show altered immune and hematopoietic phenotypes as well as defects in uterovaginal development. However, we were not able to detect any association between rs7181230 and the expression levels of *BMF* in the tissues we studied.

*HHEX* encodes a member of the homeobox family of transcription factors, many of which are involved in developmental processes. This gene has been found to be associated with type 2 diabetes by several recent GWAS [45]–[51]. The risk alleles of the diabetes-associated SNPs rs1111875 and rs5015480 are associated with low serum DHEAS concentrations although the p values (p = 0.0009 for both SNPs) didn't reach to the GWAS significance level. This is consistent with the observation in which the low serum DHEAS concentrations were associated with insulin resistance [6], [7]. The identified DHEAS-associated SNP rs2497306 is in moderate LD with rs1111875 and rs5015480 (r^2^ = 0.38). And the major allele of rs2497306 is associated with increasing serum DHEAS concentrations. The reason for the observed association is unknown. Studies showed that insulin infusion increases the metabolic clearance of DHEA and DHEAS [52], [53], resulting in decreased DHEA and DHEAS concentrations, and DHEA administration significantly enhances insulin sensitivity attenuating the age-related decline in glucose tolerance [54], partly explaining why the diabetes-associated gene is also associated with DHEAS. Interestingly, *HHEX* null mice show cardiovascular, endocrine, liver, muscle, nervous system, and metabolic phenotypes, suggesting extensive multisystem roles for the protein product of this gene. The findings could help dissect causal pathways for the observed associations between DHEAS, insulin resistance, age-related decline in glucose tolerance [54], and other age related phenotypes [55].

Three identified DHEAS-associated SNPs on chromosome 7 (Figure S2), which were independent, and 161 kb downstream (rs740160) and 370 kb upstream (rs17277546) apart from rs11761528 which is located in the middle of the region, are located in four genes - *ZKSCAN5*, *ARPC1A*, *and TRIM4/CYP3A43*. *ZKSCAN5* encodes a zinc finger protein of the Kruppel family and is expressed ubiquitously in adult and fetal tissues with the strongest expression in testis [56]. rs11761528 is located in the intron of the *ZKSCAN5* gene. It is the strongest signal we found and explains 1% of the total variance of serum DHEAS concentration alone. *ARPC1A* encodes one of seven subunits of the human Arp2/3 protein complex which has been implicated in actin polymerization and filament assembly in cells [57]. *TRIM4* encodes a member of the tripartite motif (TRIM) family whereas *CYP3A43* is another cytochrome P450 enzyme. The potential mechanisms for the association are unknown, but we found that rs17277546 is strongly associated with expression levels of *TRIM4* not *CYP3A43*, suggesting *TRIM4* is the possible candidate for DHEAS. However, rs17277546 is the best index SNP for both *CYP3A43* and *CYP3A4* genes in the pathway analysis, indicating a fine mapping in this region is needed to reveal the potential mechanism for the association. Further, the region harbours many other genes including *CYP3A7* which has been reported to increase the clearance of DHEA and DHEAS [58] and a common haplotype polymorphism in the gene has been associated with DHEAS [59], [60]. However, none of the DHEAS-associated SNPs are associated with its expression levels in the tissues we studied, and the best index SNP rs4646450 for *CYP3A7* found in our pathway analysis is in LD with rs11761528 and become non-significant in the conditional analysis.

In the pathway analysis, two DHEAS-associated SNPs (rs2185570 and rs17277546) were contained in all three pathways we found and one SNP (rs2637125) was contained in the RXR function pathway. Intriguingly, components of the xenobiotic metabolism pathway have been linked to ageing in model organisms, for example, age-associated changes in expression of genes involved in xenobiotic metabolism have been identified in rats [31], [32], up-regulation of xenobiotic detoxification genes has been observed in long-lived mutant mice [33], and adrenal xenobiotic-metabolizing activities increase with ageing in guinea pigs [34]. Furthermore, linoleic acid metabolism has also been linked to changes with ageing in rat cardiac muscle [35] and in human skin fibroblasts [36]. Taken together, these findings suggest that molecular pathways involved in ageing and longevity may also underlie DHEAS regulation, suggesting shared genetic components in both processes and corroborating a role for DHEAS as a marker of biological ageing.

In summary, this first GWAS identified eight independent SNPs associated with serum DHEAS concentrations. The related genes have various associations with steroid hormone metabolism, co-morbidities of ageing including type 2 diabetes, lymphoma, actin filament assembly, drug and xenobiotic metabolism, and zinc fingers - suggesting a wider functional role for DHEAS than previously thought.

## Methods

### Study population

Seven study samples contributed to this meta-analysis of GWA studies on serum DHEAS concentrations, comprising a total of 14,846 men and women of Caucasian origin. The consortium was made up of populations from TwinsUK (n = 4,906), Framingham Heart Study (FHS) (n = 3,183), SHIP (n = 1,832), Rotterdam Study (RS1) (n = 1,597), InCHIANTI (n = 1,182), Health ABC (n = 1,222), and GOOD (n = 924). Full details can be found in Text S1.

### DHEAS methods

Blood samples were collected from each of the study participants either after overnight fasting or non-fasting and the serum levels of DHEAS were measured by either immunoassay or liquid chromatography tandem mass spectrometry (LC-MS/MS) methods (Text S1). Because the distribution of the serum DHEAS levels was skewed, we log transformed the concentrations and the transformed data used in the subsequent analysis.

### Genotyping and imputation

Seven study populations were genotyped using a variety of genotyping platforms including Illumina (HumanHap 317k, 550k, 610k, 1M-Duo BeadChip) and Affymetrix (array 500K, 6.0). Each cohort followed a strict quality control on the genotyping data. More details on the quality control and filtering criteria can be found in Text S1. In order to increase genomic coverage and allow the evaluation of the same SNPs across as many study populations as possible, each study imputed genotype data based on the HapMap CEU Build 36. Algorithms were used to infer unobserved genotypes in a probabilistic manner in either MACH (http://www.sph.umich.edu/csg/abecasis/MACH), or IMPUTE [61]. We exclude non-genotyped SNPs with an imputation quality score <0.2 and SNPs with allele frequency <0.01 from meta-analysis.

### Statistical method

Each study performed genome-wide association testing for serum concentrations of DHEAS across approximately 2.5 million SNPs under an additive genetic model separately in men and women (Text S1). The analyses were adjusted for age. In addition, the association testing was performed in the combined men and women data with adjustment for age and sex. Studies used PLINK, GenABEL, SNPTEST, QUICKTEST, or MERLIN for the association testing. The summary results from each cohort were meta-analyzed by Z-score pooling method implemented in Metal (http://www.sph.umich.edu/csg/abecasis/metal/). We chose this method to minimize the impact of the different assays used for serum DHEAS measurements. Specifically, for each study, we converted the two-sided P value after adjustment for population stratification by the genomic control method to a Z statistic that was signed to reflect the direction of the association given the reference allele. Each Z score was then weighted; the squared weights were chosen to sum to 1, and each sample-specific weight was proportional to the square root of the effective number of individuals in the sample. We summed the weighted Z statistics across studies and converted the summary Z score to a two-sided P value. We also used I^2^ index to assess between-study heterogeneity and the inverse variance weighted method to estimate the effect size. Genome-wide significance was defined as p<5×10^−8^. The association between the DHEAS-associated SNPs and the related gene expression levels in MuTHER data were examined by mixed linear regression modelling which takes both family structure and batch effects into account. The significance was defined as p<0.006 after accounting for multiple testing (Bonferroni method, correcting 9 independent tests).

#### Pathway analysis

Meta-Analysis Gene-set Enrichment of variaNT Associations (MAGENTA) was used to explore pathway-based associations in the full GWAS dataset. MAGENTA implements a gene set enrichment analysis (GSEA) based approach, the methodology of which is described in Segrè et al [30]. Briefly, each gene in the genome is mapped to a single index SNP with the lowest P-value within a 110 kb upstream, 40 kb downstream window. This P-value, representing a gene score, is then corrected for confounding factors such as gene size, SNP density and LD-related properties in a regression model. Genes within the HLA-region were excluded from analysis due to difficulties in accounting for gene density and LD patterns. Each mapped gene in the genome is then ranked by its adjusted gene score. At a given significance threshold (95th and 75th percentiles of all gene scores), the observed number of gene scores in a given pathway, with a ranked score above the specified threshold percentile, is calculated. This observed statistic is then compared to 1,000,000 randomly permuted pathways of identical size. This generates an empirical GSEA P-value for each pathway. Significance was determined when an individual pathway reached a false discovery rate (FDR)<0.05 in either analysis. In total, 2529 pathways from Gene Ontology, PANTHER, KEGG and Ingenuity were tested for enrichment of multiple modest associations with serum DHEAS levels.

### Ethics statement

All studies were approved by local ethics committees and all participants provided written informed consent as stated in Text S1.

## Supporting Information

Figure S1 Three pathways which were associated with DHEAS. The genes which are near the DHEAS-associated SNPs are highlighted by red circles. a. Xenobiotic metabolism pathway; b. Retinoid X receptor (RXR) function pathway; c. Linoleic acid metabolism pathway; d. Legends for the pathway figures. The pathway figures were made using MetaCore from GeneGo (http://www.genego.com/metacore.php).
(TIF)

Figure S2 Regional linkage disequilibrium plots for three SNPs on chromosome 7 in one plot.
(TIF)

Table S1 87 SNPs associated with DHEAS in men, women, and combined meta-analysis with p<5×10^−8^.
(XLS)

Table S2 Pathway analysis results – list of all pathways, significant pathways, and significant genes with the best index SNPs.
(XLS)

Text S1 Supplementary Note.
(DOC)
