# Supplementary figures and images for: Eight Common Genetic Variants Associated with Serum DHEAS Levels Suggest a Key Role in Ageing Mechanisms
Source: PLoS Genet. 2011 Apr 14;7(4):e1002025. doi: 10.1371/journal.pgen.1002025 (PMC3077384; doi:10.1371/journal.pgen.1002025)

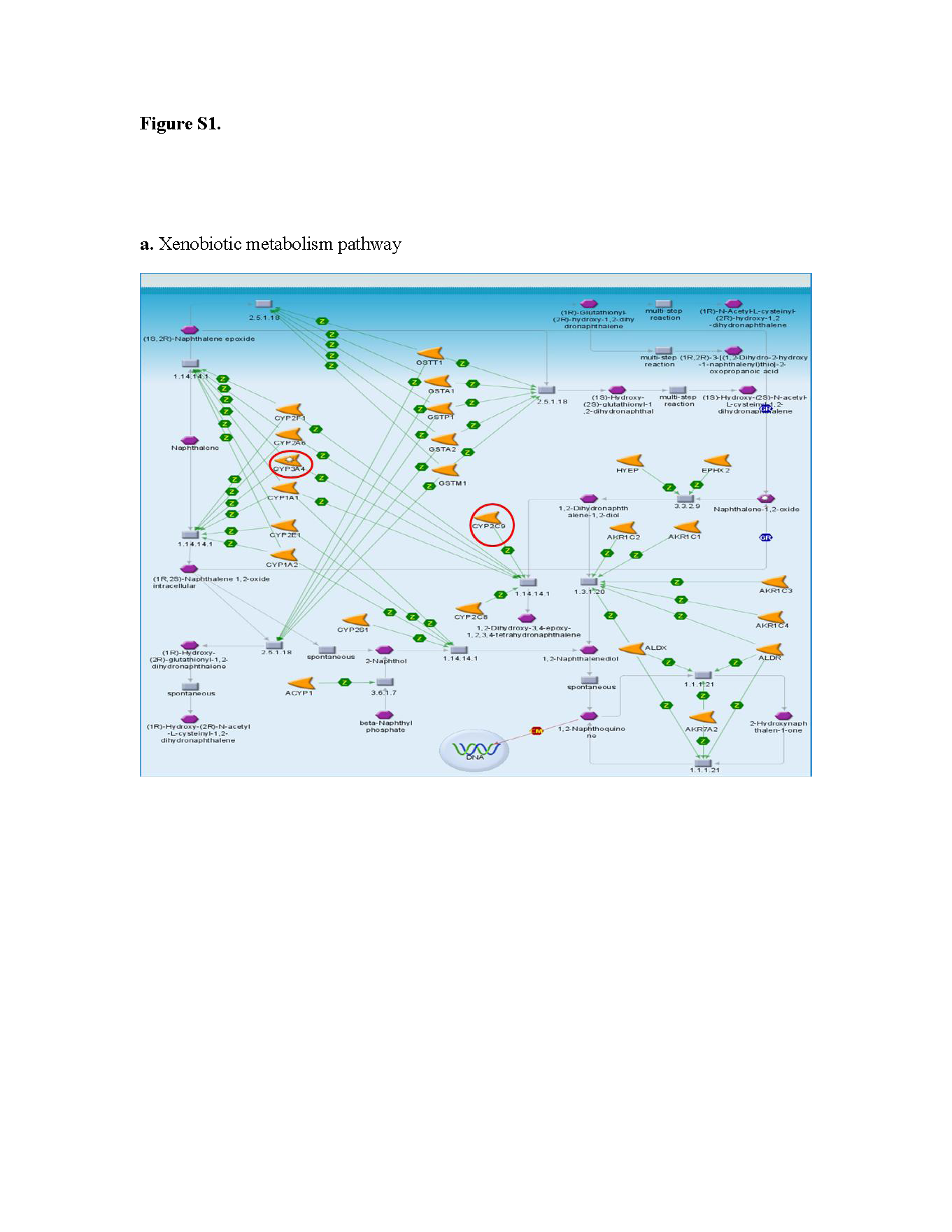

Supplement: Figure S1 — Three pathways which were associated with DHEAS. The genes which are near the DHEAS-associated SNPs are highlighted by red circles. a. Xenobiotic metabolism pathway; b. Retinoid X receptor (RXR) function pathway; c. Linoleic acid metabolism pathway; d. Legends for the pathway figures. The pathway figures were made using MetaCore from GeneGo (http://www.genego.com/metacore.php). (TIF) [file pgen.1002025.s001.tif]

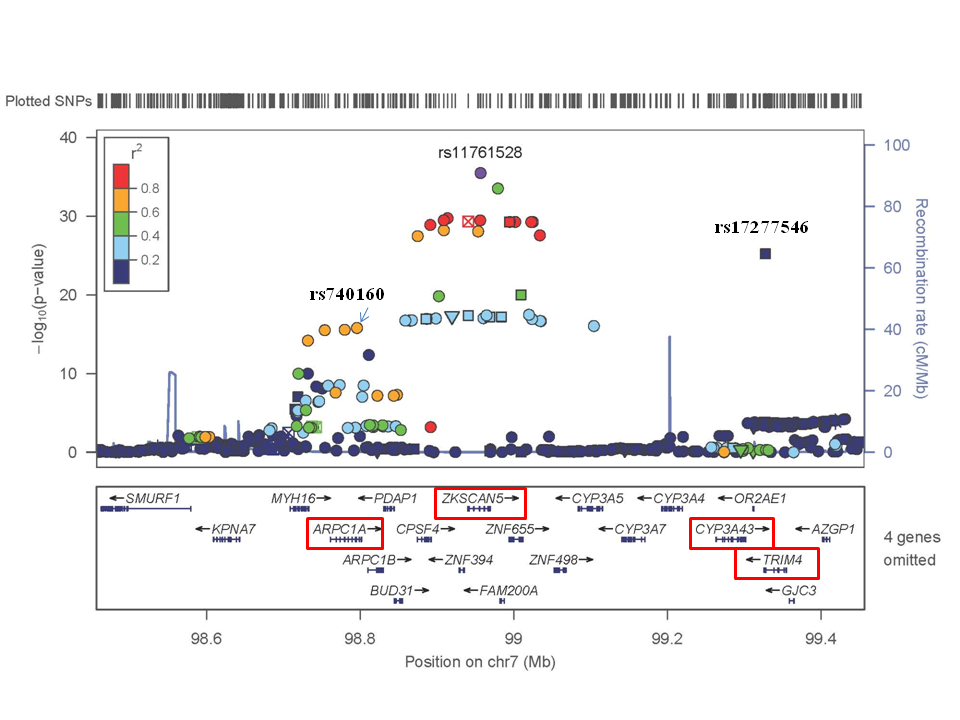

Supplement: Figure S2 — Regional linkage disequilibrium plots for three SNPs on chromosome 7 in one plot. (TIF) [file pgen.1002025.s002.tif]
