# Supplementary material for: Eight Common Genetic Variants Associated with Serum DHEAS Levels Suggest a Key Role in Ageing Mechanisms
Source: PLoS Genet. 2011 Apr 14;7(4):e1002025. doi: 10.1371/journal.pgen.1002025 (PMC3077384; doi:10.1371/journal.pgen.1002025)
Supplement: Text S1 — Supplementary Note. (DOC) [file pgen.1002025.s005.doc]

**Note S1**

**Eight common genetic variants associated with serum DHEAS levels suggest a key role in ageing mechanisms**

**G. Zhai et al.**

**Individual cohort descriptions**

**TwinsUK** is a UK-wide twin registry sample of 11,000 adults founded in 1993 with the aim to explore the genetic epidemiology of common adult diseases . The cohort has been tested to be generalizable to UK population singletons with no population stratification for a wide variety of musculoskeletal, CVD, and metabolic traits . Over 7,000 twins have attended detailed clinical examinations with a wide range of phenotypes over last 18 years. For the purpose of the current study, a total of 4906 twins had available data on serum DHEAS measurements and therefore were included in the analysis. Serum concentration of DHEAS was measured by automated electrochemiluminescence immunoassay “ECLIA” (Roche Diagnostics, Mannheim, Germany). The intra- and interassay CVs were 2.3% and 4.4%, respectively. Ethics approval was obtained from the Guy’s and St. Thomas’ Hospital Ethics Committee. Written informed consent was obtained from every participant to the study.

**Study of Health in Pomerania (SHIP)** is a longitudinal cohort study in West Pomerania, the north-east area of Germany . From the entire study population of 212,157 inhabitants living in the area, a sample was selected from the population registration offices, where all German inhabitants are registered. Only individuals with German citizenship and main residency in the study area were included. A two-stage cluster sampling method was adopted from the WHO MONICA Project Augsburg, Germany. In a first step, the three cities of the region (with 17,076 to 65,977 inhabitants) and the 12 towns (with 1,516 to 3,044 inhabitants) were selected. Further 17 out of 97 smaller towns (with less than 1,500 inhabitants) were drawn at random. In a second step, from each of the selected communities, subjects were drawn at random, proportional to the population size of each community and stratified by age and gender. Finally, 7,008 subjects aged 20 to 79 years were sampled, with 292 persons of each gender in each of the twelve five-year age strata. In order to minimize drop-outs by migration or death, subjects were selected in two waves. The net sample (without migrated or deceased persons) comprised 6,267 eligible subjects. Selected persons received a maximum of three written invitations. In case of non-response, letters were followed by a phone call or by home visits if contact by phone was not possible. The SHIP population finally comprised 4,308 participants at baseline (corresponding to a final response of 68.8%). Of the 2,116 male participants, men with missing genotype or phenotype data were excluded, as well as participants reporting chemical or surgical castration or intake of sexual hormones (anatomic-therapeutical-chemical [ATC] code G03), testosterone 5α-reductase inhibitors (G04CB), or sexual hormone antagonists (L02B). Altogether, valid data were available in 1,832 males of the SHIP baseline after exclusions. Serum DHEAS concentrations were measured from frozen serum aliquots using competitive chemiluminescent enzyme immunoassays on an Immulite 2500 analyzer (Siemens Immulite 2500, ref. L5KTW, lot 110; Siemens Healthcare Medical Diagnostics, Bad Nauheim, Germany). Measurements were carried out between December 2005 and January 2006. An aliquot of two alternating levels of a third party commercial control material (Bio-Rad Lyphochek Immunoassay Plus Control, lot 40151 and lot 40152; Bio-Rad, Munich, Germany) was included in each series in single determination. The inter-assay coefficient of variation was 14.0 % with a systematic deviation of +0.21% at the 48 μg/dl level, and 8.4% with a systematic deviation of -5.0% at the 128 μg/dl level.

**The Health, Aging, and Body Composition (Health ABC) Study** is a longitudinal cohort study consisting of 3075 initially well-functioning, community-dwelling, 70- to 79-year old, black and white men and women. Participants were identified from a random sample of white Medicare beneficiaries and all age-eligible black residents in designated zip code areas surrounding Memphis, Tennessee, and Pittsburgh, Pennsylvania. Participants were eligible if they reported no difficulty in walking one quarter of a mile, going up 10 steps without resting and performing basic activities of daily living. Participants were excluded if they reported a history of active treatment for cancer in the prior three years, planned to move out of the study area in the next three years, or were currently participating in a randomized trial of a lifestyle intervention. Baseline data, collected between April 1997 and June 1998, included an in-person interview and a clinic-based examination, with evaluation of body composition, clinical and sub-clinical diseases, and physical functioning. All participants signed informed written consent forms approved by the institutional review boards of the clinical sites. For the purpose of the current study, a total of 696 white men and 526 white women had available data on serum levels of DHEAS and therefore included in the analysis . DHEAS was determined using an automated continuous, chemiluminescent immunoassay system (IMMULITE, Diagnostics Products Corporation, Los Angeles). The DHEAS assay has a sensitivity of 1.99 μg/dL with a calibration range of 30-1000 μg/dL. Measurements of higher concentrations were obtained by dilution of the original sample, when there was sufficient remaining sample. The inter-assay coefficients of variation (CV) calculated using 5% blind duplicate samples for DHEAS assays was 27.7%. Part of the reason for the high CV, however, is that this result is highly skewed. After log transformation of the data, the CV was 10.8%.

**Rotterdam Study baseline (RS1)** is a large prospective population-based cohort study of Caucasian subjects aged 55 years and over, living in the Ommoord district of Rotterdam, the Netherlands. The study was designed to investigate the incidence and determinants of chronic disabling diseases in the elderly. Rationale and design have been described previously . All 10,275 inhabitants aged 55 years and over were invited for baseline examination between August 1990 and June 1993. Of those, 7,983 participated. Among the subjects living independently, the overall response rate was 77 percent for home interview and 71 percent for examination in the research centre, where anthropometric characteristics and bone mineral density were measured, and blood samples were taken. The Rotterdam Study was approved by the medical ethics committee of the Erasmus University Medical School, and written informed consent was obtained from each subject. The current study is based on 740 men and for 857 women for whom genome-wide genotype data and DHEAS concentrations were available. Non-fasting blood samples were drawn by venipuncture at the baseline examination in the research center between 0830 and 1600 h. For the collection of plasma, blood was sampled in 5-ml tubes containing 0.5-ml sodium citrate solution. Platelets were removed by centrifugation, and the samples were stored at –80 C until hormone measurements. The period of storage of frozen serum varied from 7.5–12.5 yr. Plasma levels of DHEAS were estimated in 12 separate batches of samples using coated tube RIA, purchased from Diagnostic Systems Laboratories, Inc. (Webster, TX). Because of the relatively small volumes of plasma available, all values reported are single sample estimations. Intra-assay coefficient of variation, determined on the basis of duplicate results of internal quality control pools with three different levels of DHEAS, was below 15%. The inter-assay variations for DHEAS was 30%, therefore, we multiplied all concentrations within a batch with a factor, which made results for the internal quality control pools comparable. This reduced inter-assay variation to 10%. This was considered justified because the relative differences in the results for the high and middle internal quality control pools per batch were comparable, as evidenced by a significant correlations between these results (p<0.01).

**The Framingham Heart Study (FHS)** is a prospective cohort study initiated in 1948 to study determinants of cardiovascular disease. The Original Cohort comprised 5209 men and women and in 1971, 5124 Offspring of the Original Cohort participants and Offspring spouses, aged 5 to 70 years, were enrolled into the Framingham Offspring Study. Offspring participants have been examined approximately every 4 to 8 years . At each examination participants undergo a medical history interview, physical examination, lifestyle questionnaires and laboratory assessment of risk factors. In the 1990s, DNA was obtained for genetic studies from surviving Offspring participants. Serum samples from Offspring exam 4 (1987-1991) were used in the measurement of DHEAS levels. If data was missing at exam 4 then DHEAS values from exam 3 (1984-1987) were used. There were 1571 men and 1612 women with genome-wide genotyping and DHEAS levels available for this study. DHEAS levels were measured using radioimmunoassay (Diagnostic Products Corp, Los Angelos, CA) with an interassay coefficient of variation of 11% as previously described . The Institutional Review Board of the Boston University Medical Center approved the content of all FHS examinations and all participants provided written informed consent.

**The Gothenburg Osteoporosis and Obesity Determinants (GOOD) Study** was initiated to determine both environmental and genetic factors involved in the regulation of bone and fat mass. Male study subjects were randomly identified in the greater Gothenburg area in Sweden using national population registers, contacted by telephone, and invited to participate. To be enrolled in the GOOD study, subjects had to be between 18 and 20 years of age. There were no other exclusion criteria, and 49% of the study candidates agreed to participate (n = 1,068) . The study was approved by the ethics committee at the University of Gothenburg. Written and oral informed consent was obtained from all study participants. Serum DHEAS levels were measured from frozen serum aliquots using a validated liquid chromatography tandem mass spectrometry technique, as previously described . The assay has a sensitivity of 0.20 μmol/L, with an intra-assay CV of 5.2% and an inter-assay CVof 6.3%. After excluding subjects with missing genotype or phenotype data as well as participants reporting intake of glucocorticoids, 924 subjects were included in the analysis.

**The InCHIANTI study** is a population-based epidemiological study aimed at evaluating factors that influence mobility in the older population living in the Chianti region of Tuscany, Italy. Details of the study have been previously reported . Briefly, 1616 residents were selected from the population registry of Greve in Chianti (a rural area; 11,709 residents with 19.3% of the population greater than 65 years of age) and Bagno a Ripoli (Antella village near Florence; 4704 inhabitants, with 20.3% greater than 65 years of age). The participation rate was 90% (n= 1182) and participants ranged between 21–95 years of age. The study protocol was approved by the Italian National Institute of Research and Care of Aging Institutional Review.

**Genotyping and Quality Control Methods**

**TwinsUK:** Two sets of the samples (TwinsUK-I and TwinsUK-II) derived from the TwinsUK were genotyped. The first set – TwinsUK-I, was genotyped using the Infinium assay (Illumina, San Diego, USA) with four fully compatible SNP arrays as previously described . Normalised intensity data were pooled and genotypes called on the basis of the Illluminus algorithm . No calls were assigned if the most likely call was less than a posterior probability of 0.95. Validation of pooling was done by visual inspection of 100 random, shared SNPs for overt batch effects; none were observed. Quality checks similar to those for the WTCCC Study were applied. The second set – TwinsUK-II, were typed with the Infinium 610k assay (Illumina, San Diego, USA) at two different centres, namely the Centre for Inherited Diseases Research (USA) and the Wellcome Trust Sanger Institute. The same pooling procedure used for the TwinsUK-I was applied to the TwinsUK-II. Further, we excluded SNPs that had a low call rate (<95%), Hardy-Weinberg p values < 10^−4^ and minor allele frequencies < 1%. We also removed subjects if the sample call rate was less than 95%, autosomal heterozygosity was outside the expected range, genotype concordance was over 97% with another sample and the sample was of lesser call rate, non-Caucasian ancestry either self-identified or identified by cluster analysis in STRUCTURE or multidimensional scaling by comparison to the three HapMap phase 2 reference populations (CEU, YRI, CHB+JPT), or unexplained relatedness (estimated proportion of allele shared identical by descent >0.05) to >120 other samples where genotyping failed for >2 % of SNPs . The overall genotyping efficiency of the GWA was 98.7 %. Imputation of genotypes was carried out using the software IMPUTE for both TwinsUK-I and TwinsUK-II.

**SHIP** samples were genotyped using the Affymetrix Human SNP Array 6.0. Hybridisation of genomic DNA was done in accordance with the manufacturer’s standard recommendations. The genetic data analysis workflow was created using the Software InforSense. Genetic data were stored using the database Caché (InterSystems). Genotypes were determined using the Birdseed2 clustering algorithm. For quality control purposes, several control samples were added. On the chip level, only subjects with a genotyping rate on QC probesets (QC callrate) of at least 86% were included. All remaining arrays had a sample callrate > 92%. Samples that were detected as duplicates by IBS and samples with reported vs. genotyped gender mismatch were excluded from the analysis. The overall genotyping efficiency of the GWA was 98.55 %. Imputation of genotypes in SHIP was performed with the software IMPUTE v0.5.0 based on HapMap II.

**Health ABC:** Genomic DNA was extracted from buffy coat collected using PUREGENE® DNA Purification Kit during the baseline exam. In 2009, genotyping was performed by the Center for Inherited Disease Research (CIDR) using the Illumina Human1M-Duo BeadChip system. Samples were excluded from the dataset for the reasons of sample failure, genotypic sex mismatch, and first degree relative of an included individual based on genotype data. Genotyping was successful in 2,802 individuals (1663 Caucasians and 1139 African Americans). Genotypes were available on 914263 high quality SNPs before imputation.

**RS1:** The Rotterdam Study samples were genotyped using the Illumina Infinium HumanHap550 Beadchip The following sample QC criteria were applied in the GWAS of RS-I, sample call rate ≥97.5%, gender mismatch with typed X-linked markers, evidence for DNA contamination in the samples using the mean of the autosomal heterozygosity >0.33, exclusion of duplicates or first-degree relatives identified using IBS probabilities, exclusion of outliers (three SD away from the population mean) using multi-dimensional scaling (MDS) analysis with four PCAs. The inclusion criteria for SNPs for imputation were the following: Minor Allele Frequency ≥ 1%, SNP-callrate ≥ 97.5% and HWE-pvalue ≥ 10^-6^. Genotypes were imputed for all polymorphic SNPs (minor allele frequency >0.01) using the MACH software, based upon phased autosomal chromosomes of the HapMap CEU Phase II panel (release 22, build 36), orientated on the positive strand. Imputation QC metrics from MACH were used for filtering out SNPs with low-quality data.

**FHS:** Genotyping was obtained through the FHS SHARe (SNP Health Association Resource) project on all Framingham Study participants with DNA using the Affymetrix 500K mapping array in addition to the Affymetrix 50K supplemental array (<http://www.ncbi.nlm.nih.gov/projects/gap/cgi-bin/study.cgi?study_id=phs000007.v11.p5> ). The following sample level exclusions were used: participant call rate <97%, a per subject heterozygosity ±5 standard deviations away from the mean, or a per subject large Mendelian error rate. SNPs were filtered based on the following quality control parameters: MAF ≥0.01, HWE p>10^-6^, callrate >0.97, *mishap* *test* of non-random missingness p>10^-9^, and ≤100 Mendelian errors. MACH (version 1.0.15) was used to impute all autosomal SNPs on HapMap with the reference panel derived from the publicly available phased haplotypes from HapMap (release 22, build 36, CEU population). From a total of 534,982 genotyped autosomal SNPs, 378,163 SNPs that were present on HapMap and that passed quality control measures in the FHS sample were used for imputation. Details of the genotyping quality control procedures and imputation have been previously reported .

**The GOOD** samples were genotyped using the Illumina HumanHap610 Quad arrays at the Genetic Laboratory, Department of Internal Medicine, Erasmus Medical Center, Rotterdam, the Netherlands. Genotypes were called using the BeadStudio calling algorithm. Genotypes from 938 individuals passed the sample quality control criteria [exclusion criteria: sample call rate < 97.5%, gender discrepancy with genetic data from X-linked markers, excess autosomal heterozygosity > 0.33 ~ FDR < 0.1%, duplicates and/or first degree relatives identified using IBS probabilities (> 97%), ethnic outliers (3 SD away from the population mean) using multi-dimensional scaling analysis with four principal components]. Across 22 duplicate samples, genotype concordance exceeded 99.9%. Genotypes were imputed for all polymorphic SNPs (521,160 with MAF ≥ 1%, SNP call rate ≥ 98% and HWE p value ≥ 10^-6^) using the MACH software, based upon phased autosomal chromosomes of the HapMap CEU Phase II panel (release 22, build 36), orientated on the positive strand.

**InCHIANTI samples** were genotyped using the Illumina HumanHap 550k beadchip. Strict QC criteria were applied to genotyped SNPs before imputation: Sample/SNP callrate >98%, MAF > 1%, HWE P < 1x10^-4^. There were 85 parent-offspring pairs, 6 sib-pairs and 2 half-sibling pairs documented. We investigated any further familial relationships using IBD of 10,000 random SNPs using RELPAIR and uncovered 1 parent offspring, 79 siblings and 13 half-sibling . We utilized the correct family structure inferred from genetic data for all analyses.

**Genome-wide expression data from the Multiple Tissue Human Expression Resource (MuTHER)** (<http://www.muther.ac.uk/>): The MuTHER resource includes LCLs, skin and adipose tissue derived simultaneously from a subset of well-phenotyped healthy female twins of the TwinsUK cohort. Whole-genome expression profiling of the samples, each with either two or three technical replicates, were performed using the Illumina Human HT-12 V3 BeadChips (Illumina Inc) according to the protocol supplied by the manufacturer. Log2 transformed expression signals were normalized separately per tissue as follows: quantile normalization was performed across technical replicates of each individual followed by quantile normalization across all individuals. The participants of the MuTHER were also genotyped as described in the TwinsUK genotyping section above. The number of samples with genotypes and expression values per tissue was 778 LCL, 667 skin and 776 adipose, respectively, and used in the functional analysis.

**Statistical methods:**

**TwinsUK**: Because of the relatedness in the TwinsUK cohort, we utilized the GenABEL software package which is designed for GWAS analysis of family-based data by incorporating pair-wise kinship matrix calculated using genotyping data in the polygenic model to correct relatedness and hidden population stratification. The score test implemented in the software was used to test the association between a given SNP and DHEAS with adjustment for age as a covariate.

**Health ABC:** Linear regression was used to fit an additive model relating the imputed genotypic dose (0 to 2 copies of the coded allele) to the outcome (log transformed DHEAS). Covariate adjustments were made for age, gender, study site, and the first principal component obtained from an analysis of population stratification. A stratified analysis was also performed with this model being fit separately in men and women. Linear regression models were fit with the ‘lm’ function in R (version 2.9.2).

**RS1:** Statistical analysis of imputed data was performed using MACH2QTL implemented in GRIMP . Allelic tests were performed adjusting for age (and gender in the combined analysis) as covariates.

**FHS:** Linear mixed effects models were used to fit an additive genetic model relating the imputed genotypic dose (0 to 2 copies of the coded allele) to log transformed DHEAS. Covariate adjustments were made for age and for age and gender in the combined analysis. Linear mixed effects regression models were fit with the ‘lmekin’ function in R kinship package.

**SHIP:** DHEAS values were available for men only. Association testing was using an additive genetic model adjusted for age and taking the uncertainties for imputed genotypes into account. Genome-wide association tests were performed using QUICKTEST v0.95 with the score-method. (http://toby.freeshell.org/software/quicktest.shtml).

**GOOD:** Association testing for imputed SNPs was carried out using MACH2QTL implemented in GRIMP . Uncertainty in genotype prediction was accounted for by utilizing the dosage information from MACH.

**InCHIANTI:** Imputation was performed using MACH. Additive genetic test statistics were produced by MACH2QTL. Age and sex were included as covariates in the appropriate analyses.

**References**

1. Spector TD, Williams FM (2006) The UK Adult Twin Registry (TwinsUK). Twin Res Hum Genet 9:899-906

2. Andrew T, Hart DJ, Snieder H, de Lange M, Spector TD, MacGregor AJ (2001) Are twins and singletons comparable? A study of disease-related and lifestyle characteristics in adult women. Twin Res 4:464-477

3. Volzke H, Alte D, Schmidt CO, et al. (2010) Cohort Profile: The Study of Health in Pomerania. Int J Epidemiol

4. Friedrich N, Volzke H, Rosskopf D, Steveling A, Krebs A, Nauck M, Wallaschofski H (2008) Reference ranges for serum dehydroepiandrosterone sulfate and testosterone in adult men. J Androl 29:610-617

5. Morsink LF, Vogelzangs N, Nicklas BJ, et al. (2007) Associations between sex steroid hormone levels and depressive symptoms in elderly men and women: results from the Health ABC study. Psychoneuroendocrinology 32:874-883

6. Hofman A, Breteler MM, van Duijn CM, Krestin GP, Pols HA, Stricker BH, Tiemeier H, Uitterlinden AG, Vingerling JR, Witteman JC (2007) The Rotterdam Study: objectives and design update. Eur J Epidemiol 22:819-829

7. Feinleib M, Kannel WB, Garrison RJ, McNamara PM, Castelli WP (1975) The Framingham Offspring Study. Design and preliminary data. Prev Med 4:518-525

8. Kannel WB, Feinleib M, McNamara PM, Garrison RJ, Castelli WP (1979) An investigation of coronary heart disease in families. The Framingham offspring study. Am J Epidemiol 110:281-290

9. Amin S, Zhang Y, Sawin CT, Evans SR, Hannan MT, Kiel DP, Wilson PW, Felson DT (2000) Association of hypogonadism and estradiol levels with bone mineral density in elderly men from the Framingham study. Ann Intern Med 133:951-963

10. Lorentzon M, Swanson C, Andersson N, Mellstrom D, Ohlsson C (2005) Free testosterone is a positive, whereas free estradiol is a negative, predictor of cortical bone size in young Swedish men: the GOOD study. J Bone Miner Res 20:1334-1341

11. Vandenput L, Labrie F, Mellstrom D, et al. (2007) Serum levels of specific glucuronidated androgen metabolites predict BMD and prostate volume in elderly men. J Bone Miner Res 22:220-227

12. Ferrucci L, Bandinelli S, Benvenuti E, Di Iorio A, Macchi C, Harris TB, Guralnik JM (2000) Subsystems contributing to the decline in ability to walk: bridging the gap between epidemiology and geriatric practice in the InCHIANTI study. J Am Geriatr Soc 48:1618-1625

13. Richards JB, Rivadeneira F, Inouye M, et al. (2008) Bone mineral density, osteoporosis, and osteoporotic fractures: a genome-wide association study. Lancet 371:1505-1512

14. Teo YY, Inouye M, Small KS, Gwilliam R, Deloukas P, Kwiatkowski DP, Clark TG (2007) A genotype calling algorithm for the Illumina BeadArray platform. Bioinformatics (Oxford, England) 23:2741-2746

15. (2007) Genome-wide association study of 14,000 cases of seven common diseases and 3,000 shared controls. Nature 447:661-678

16. Pritchard JK, Stephens M, Donnelly P (2000) Inference of population structure using multilocus genotype data. Genetics 155:945-959

17. Purcell S, Neale B, Todd-Brown K, et al. (2007) PLINK: a tool set for whole-genome association and population-based linkage analyses. Am J Hum Genet 81:559-575

18. Marchini J, Howie B, Myers S, McVean G, Donnelly P (2007) A new multipoint method for genome-wide association studies by imputation of genotypes. Nat Genet 39:906-913

19. Psaty BM, O'Donnell CJ, Gudnason V, Lunetta KL, Folsom AR, Rotter JI, Uitterlinden AG, Harris TB, Witteman JC, Boerwinkle E (2009) Cohorts for Heart and Aging Research in Genomic Epidemiology (CHARGE) Consortium: Design of prospective meta-analyses of genome-wide association studies from 5 cohorts. Circ Cardiovasc Genet 2:73-80

20. Epstein MP, Duren WL, Boehnke M (2000) Improved inference of relationship for pairs of individuals. Am J Hum Genet 67:1219-1231

21. Aulchenko YS, Ripke S, Isaacs A, van Duijn CM (2007) GenABEL: an R library for genome-wide association analysis. Bioinformatics (Oxford, England) 23:1294-1296

22. Estrada K, Abuseiris A, Grosveld FG, Uitterlinden AG, Knoch TA, Rivadeneira F (2009) GRIMP: a web- and grid-based tool for high-speed analysis of large-scale genome-wide association using imputed data. Bioinformatics (Oxford, England) 25:2750-2752
